# Identification and Expression Analysis of the Isopentenyl Transferase (IPT) Gene Family under Lack of Nitrogen Stress in Oilseed (*Brassica napus* L.)

**DOI:** 10.3390/plants12112166

**Published:** 2023-05-30

**Authors:** Jingdong Chen, Heping Wan, Wenhui Zhu, Xigang Dai, Yi Yu, Changli Zeng

**Affiliations:** 1 College of Life Science, Jianghan University, Wuhan 430056, China; cjd19951226@126.com (J.C.);; 2 College of Life Science, Fujian Agriculture and Forestry University, Fuzhou 350002, China

**Keywords:** *Brassica napus*, IPT gene family, nitrogen, abiotic stress tolerance

## Abstract

*BnIPT* gene family members in *Brassica napus* and analyzing their expression under different exogenous hormones and abiotic stress treatments to provide a theoretical basis for clarifying their functions and molecular genetic mechanisms in nitrogen deficiency stress tolerance of *B. napus*. Using the Arabidopsis IPT protein as the seed sequence, combined with the IPT protein domain PF01715, 26 members of the *BnIPT* gene family were identified from the whole genome of the rape variety ZS11. Additionally, the physicochemical properties and structures, phylogenetic relationships, synteny relationships, protein–protein interaction network, and gene ontology enrichment were analyzed. Based on transcriptome data, the expression patterns of the *BnIPT* gene under different exogenous hormone and abiotic stress treatments were analyzed. We used the qPCR method to identify the relative expression level of *BnIPT* genes that may be related to the stress resistance of rapeseed in transcriptome analysis under normal nitrogen (N: 6 mmol·L^−1^) and nitrogen deficiency (N: 0) conditions and analyzed its effect on rapeseed under nitrogen deficiency stress role in tolerance. In response to nitrogen deficiency signals, the *BnIPT* gene showed a trend of up-regulation in shoots and down-regulation in roots, indicating that it may affect the process of nitrogen transport and redistribution to enhance the stress resistance of rapeseed to respond to the nitrogen deficiency stress. This study provides a theoretical basis for clarifying the function and molecular genetic mechanism of the *BnIPT* gene family in nitrogen deficiency stress tolerance in rape.

## 1. Introduction

Phytohormones, as growth regulators synthesized by plants themselves, with relatively simple chemical structures and a small amount of synthesis, play important physiological regulatory functions in the process of plant growth and development [1]. As one of the main plant hormones, cytokinins (CKs) are involved in the regulation of plant growth and development. At the same time, they have various links with other hormones, which both restrict and promote each other, maintaining the dynamic balance of hormone levels as well as regulating plant growth and development [2].

It was shown that CKs could not only increase the yield of crops but also improve the stress resistance of crops [3]. The naturally occurring active CKs are N^6^-isopentenyl adenine derivatives, which are synthesized in root and shoot apical meristems [4]. According to the structure of the side chain, CKs can be divided into isoprenoid-type CKs and aromatic-type CKs. The isoprenoid forms of CKs include the isopentenyl adenine (zeatin) forms, trans-zeatin forms, and cis-zeatin forms [5]. There are currently two biosynthetic pathways of CKs known in plants, tRNA degradation and de novo synthesis. The earliest study believed that the degradation of tRNA was the initial pathway of CK synthesis [6]. The cis-zeatin released by the degradation of tRNA is catalyzed by cis-trans isomerase and converted into highly active trans-zeatin. However, later research results showed that there was almost no conversion between cis-zeatin and trans-zeatin [7]. Therefore, the low conversion rate of tRNA cannot meet the synthesis demand of a large number of CKs in plants, indicating that the tRNA degradation pathway is only a secondary pathway of CK biosynthesis. It has been demonstrated that de novo synthesis is the major pathway for CK biosynthesis in plants, including the AMP pathway, the ATP/ADP pathway, and the bypass pathway [8].

The research on the synthetic pathway of CKs started from the *AtIPT* genes of the model plant, *Arabidopsis thaliana*. The *AtIPTs* in Arabidopsis are encoded by a small multigene family with nine family members (*AtIPT1-AtIPT9*). Bioinformatics analysis showed that *AtIPT 2* and *AtIPT 9* belonged to the tRNA-IPT type, and the other seven genes, namely *AtIPT 1* and *AtIPT 3–AtIPT 8*, belonged to the ATP-IPT type, which had higher homology acid sequences with the bacterial ATP/ADP-IPT proteins [9,10]. Isopentenyl transferase (IPT) is the first rate-limiting enzyme that catalyzes the biosynthesis of CKs. It plays an important role in plant response to adversity stress, such as salt stress [11], drought stress [12], high-temperature stress [13], and low-temperature coercion [14].

CKs are one of the main regulators of plant growth and stress resistance and can regulate the process of nitrogen uptake, transport, and redistribution in higher plants [15]. For example, under moderate soil drought conditions, rice could enhance the reactivation process of nitrogen from source to sink by redistributing the concentration of CKs after anthesis [16]. *OlIPTs* in wild rice *Oryza longistaminata* could respond to nitrogen accumulation. *OlIPT4*, *OlIPT5*, and *OlIPT8* accumulated in the rhizome node that includes an axillary bud under 6 h of nitrogen application compared to nitrogen-deficient conditions, while *OlIPT4* accumulates from 2 h after application of 2 mmol·L^−1^ ammonium nitrate [17].

Oilseed rapes are one of the most important oil crops in the world, among which kale-type rapes (*Brassica napus* L.) have the largest planting area. *B. napus*, as an important cash crop, in addition to its oil-rich seeds, can be used as an important source of edible oil for humans and industrial raw materials [18]. Meanwhile, seedcake of it can also be used as feed to provide an important source of protein for livestock [19]. In recent years, as a beautiful ornamental flower, *B. napus* has also been widely planted to develop ecotourism industries around the world, bringing huge economic benefits to agricultural production [20]. Nitrogen (N) is one of the macroelements necessary for plant growth, which plays an important role in the metabolism, yield, and quality of plants [21]. *B. napus* is generally considered to have a large demand for nitrogen nutrients during growth and development, but its nitrogen efficiency is relatively low [22]. Therefore, in agricultural production, people have to ensure the normal growth of rape by applying large amounts of nitrogen fertilizer [23]. N deficiency also affects the normal growth and development of *B. napus*, making it less productive [24].

With the development of modern technology, bioinformatics has gradually matured and is able to continuously reduce the cost of generating a large amount of sequence information. Currently, bioinformatics is playing an increasingly important role in agroinformatics. Bioinformatics is also playing an increasingly important role in agroinformatics [25]. The bioinformatics of *B. napus* mainly focuses on the bioinformatics analysis of related enzymes, proteins, hormones, and other related genes, and evaluates the traits of genes through bioinformatics analysis (including sequence characteristics, structure function and cluster analysis, etc.) [26], reveal the mechanism of action of related genes and pave the way for further in-depth research in the future.

Although the role of *IPTs* in affecting plant CK levels and resistance to abiotic stress has been extensively studied in Arabidopsis and other crops [11,12,13,14], there is less research on the expression of these genes in *B. napus* under nitrogen-deficiency stress. The purpose of this paper is to investigate the distribution of *BnIPTs* in *B. napus* chromosomes, to study their role in resisting abiotic stresses in oilseed rape, especially under nitrogen deficiency conditions, and to provide a basis for the screening and creation of nitrogen deficiency-tolerant germplasms. In this paper, genome-wide identification of *BnIPT* gene family members in oilseed rape was performed, and systematic analyses of protein characterization, gene structure, phylogeny, and collinearity were predicted. The responses of *BnIPTs* expression levels in leaves and roots under different adversities (salt, drought, cold, heat, osmotic) and different exogenous hormones (cytokinin, growth hormone, gibberellin, abscisic acid, trans-zeatin, oleuropein lactone) treatments were investigated by RNA-seq information. Additionally, by setting two levels of nitrogen, lack of nitrogen (N: 0) and normal nitrogen (N: 6 mmol·L^−1^), the expression of *BnIPTs* in the sprouts of rapeseed at the germination stage was tested, which provided evidence that *BnIPTs* responded to nitrogen metabolism signals. The research results provide a theoretical basis for further analysis of the functional roles of these genes in the process of nitrogen metabolism in rapeseed.

## 2. Results and Analysis

### 2.1. Identification and Chromosomal Localization of the BnIPT Gene Family

**Figure 1:**
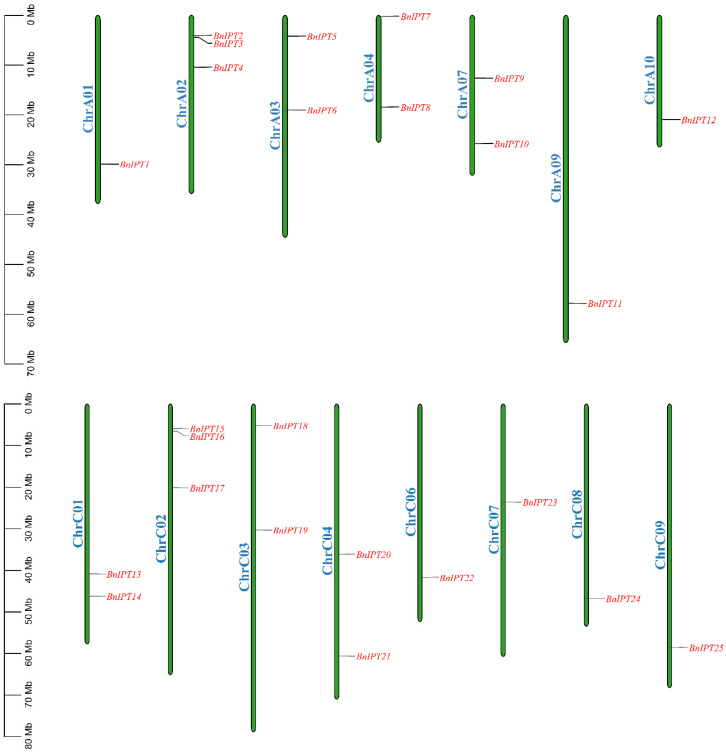
Chromosomal location of the *BnIPTs* in the *B. napus* genome.

The protein sequences of nine Arabidopsis IPT genes were downloaded from TAIR as seed sequences, and the whole genome protein sequence file of oilseed rape was used as a database with the e value set to 1 × 10^−10^ to identify the possible BnIPT members by BLASTP. The IPPT domain HMM matrix of IPT protein was downloaded from the Pfam website, and the BnIPT hypothetical proteins with this structure were searched for with the HMMER software. The hypothetical proteins identified by both BLAST and HMMER were used to remove incomplete domain sequences by the NCBI-CDD tool, and 26 BnIPT members were finally determined, which were named *BnIPT1–BnIPT26* according to the orders of genes on the chromosome. A total of 25 of these members were localized to 15 identified chromosomes (Figure 1), and 1 was localized to pseudochromosome Scaffold0026. Among them, three *BnIPTs* were mapped to each of chromosomes A02 and C02; two *BnIPTs* were mapped to each of chromosomes A03, A04, A07, C01, C03, and C04; and one *BnIPT* was mapped to each of chromosomes A01, A10, C06, C07, C08, and C09. None of the *BnIPTs* were detected on chromosomes A05, A06, and C05.

### 2.2. Prediction of Physicochemical Properties, Structure, and Subcellular Localization of BnIPT Protein

By prediction analysis, the number of amino acids (AAs) encoded by BnIPT proteins is between 319 and 464, the molecular weight (MW) of the proteins ranged from 35.76 to 52.46 kDa, and the theoretical isoelectric point (PI) was between 5.40 and 9.07. The grand average of hydropathicity (GRAVY) ranged from −0.562 to −0.137, indicating that they exhibited hydrophilicity (Table S1). The subcellular localization prediction results showed that all BnIPT proteins were predicted to exist in the chloroplast, BnIPT4, BnIPT8, BnIPT15, BnIPT16, BnIPT17, and BnIPT22 might also exist in the cytoplasm, and BnIPT5, BnIPT9, BnIPT15, BnIPT16, and BnIPT22 might also exist in mitochondria; BnIPT3 may also exist in the cell membrane and nucleus.

Protein secondary structure prediction showed that all BnIPT proteins contained three structures: α-helix (Hh), extended backbone (Ee), and random coil (Cc) (Table 1). Among them, Hh occupied most of the structural types, with a ratio of 42.82% to 52.80%; Cc occupied a medium proportion, with a ratio of 36.68% to 43.39%; and Ee occupied the smallest proportion, with a ratio of 8.44% to 13.79%. The prediction of the tertiary structure showed that the BnIPT proteins were mainly composed of Hh and Cc, which was consistent with the prediction of the protein secondary structure based on homology modeling (Figure S1).

The tertiary structure models of most BnIPT proteins were highly similar, with typical Hh and Cc structures in the model. At the same time, it was found that the tertiary structure models of BnIPT3, BnIPT5, BnIPT8, BnIPT9, BnIPT16, BnIPT18, BnIPT19, BnIPT21, and BnIPT26 also had Hh and Cc structures, but they were different from other protein models. Considering the low confidence in the prediction of some structures in their models (the redder the color of the structure in the graph, the lower the confidence; on the contrary, the bluer the color, the higher the confidence), it can be tentatively judged that the BnIPT proteins have tertiary structural similarity.

### 2.3. Phylogenetic Analysis of the IPT Family in B. napus

**Figure 2:**
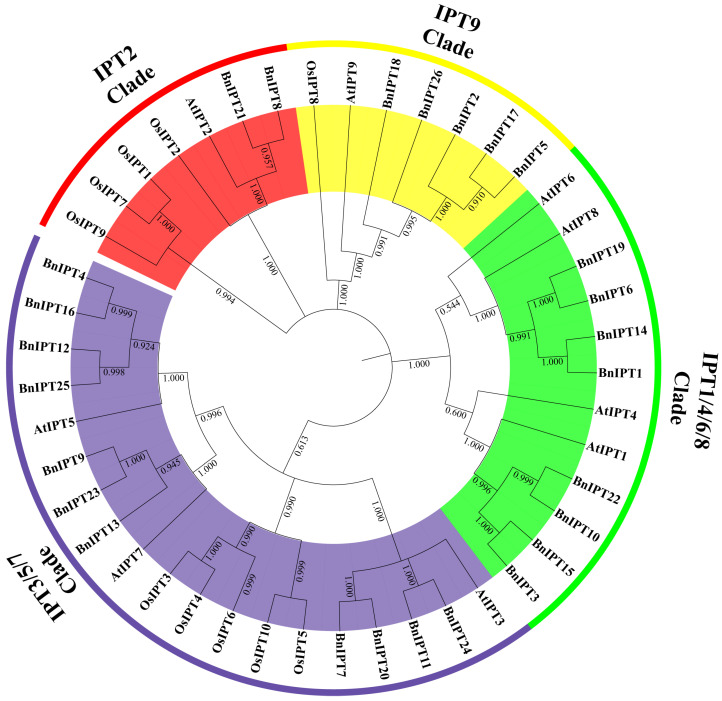
Phylogenetic tree of IPT proteins in *B. napus*, *A. Thaliana*, and *O. sativa*.

To explore the phylogenetic relationships among IPT proteins, 26 IPT protein members from oilseed rape, 9 from Arabidopsis, and 10 from rice were combined to form the phylogenetic tree. Based on the genetic relationship between them and according to the clade position of the AtIPT protein, the IPT protein is divided into four clades: IPT2, IPT9, IPT1/4/6/8, and IPT3/5/7 (Figure 2). The number of proteins within each clade was different, with the largest number of proteins clustered into the IPT3/5/7 branch with 19, followed by the IPT1/4/6/8 branch with 12, and the smallest number of IPT2 and IPT9 with only 7.

### 2.4. Analysis of Rape IPT Family Structure, Conserved Motifs, and Promoter Cis-Acting Elements

**Figure 3:**
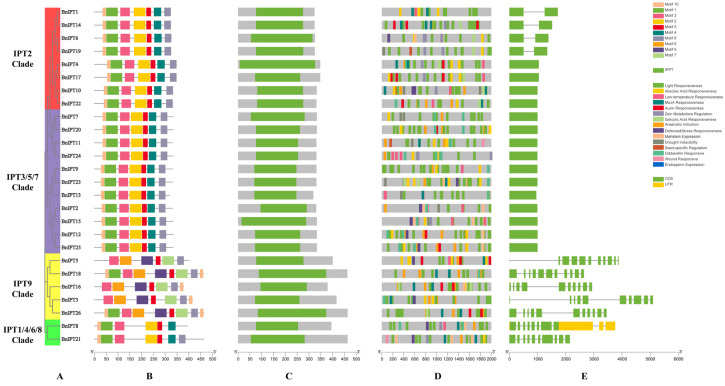
Gene structure analysis of *IPT* family in *B.napus*. (**A**) Phylogenetic tree of BnIPT family. (**B**) Conserved motifs of BnIPT family proteins. (**C**) Structure of BnIPT family protein Pfam. (**D**) Promoter cis-acting element of *BnIPT* family. (**E**) The mRNA structure encoded by the *BnIPT* family.

Analysis of the conserved motifs of the BnIPT proteins showed that Motif 3 and Motif 5 were present in all BnIPT proteins (Figure 3B). Among them, the IPT9 Clade was the most special; only two proteins contained Motif 1 and Motif 10, and all the IPT9 Clade proteins did not contain Motif 2 and Motif 4, while all the proteins other three clades contained these motifs. Additionally, only all the proteins in the IPT9 Clade contained Motif 7, Motif 8, and Motif 9, while Motif 6 was present in all BnIPT proteins except for the BnIPT8 protein (belonging to IPT1/4/6/8 Clade). The protein structural features of BnIPT were identified, and all proteins were found to contain the IPPT structural domain (Figure 3C). Analysis of promoter cis-acting elements revealed that *BnIPTs* might have a response mechanism in response to stress and phytohormone signals, and a variety of cis-acting elements were identified in this study (Figure 3D). The number of light-responsive elements was as high as 328, far more than other cis-acting elements. In addition, *BnIPT12* contained the largest number of 34 cis-acting elements, and it is speculated that it may play an important role in the growth and development of rapeseed. The structure of *BnIPT*-encoded mRNAs was investigated, and it was found that they contained 1~11 CDS (coding sequence) regions (Figure 3E), and the *BnIPT*-encoded mRNA was the most special, also containing 3 UTRs (untranslated regions).

### 2.5. Collinearity Analysis of IPTs

**Figure 4:**
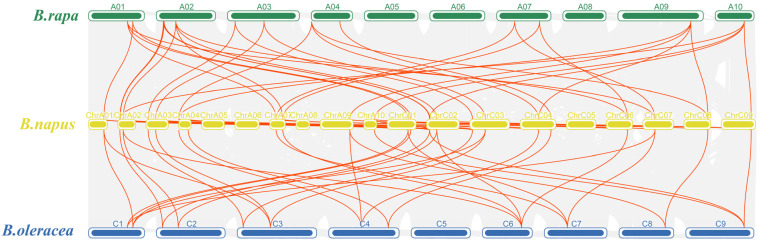
Collinearity of *IPT* genes in *B. napus*, *B. rapa*, and *B. oleracea*.

**Figure 5:**
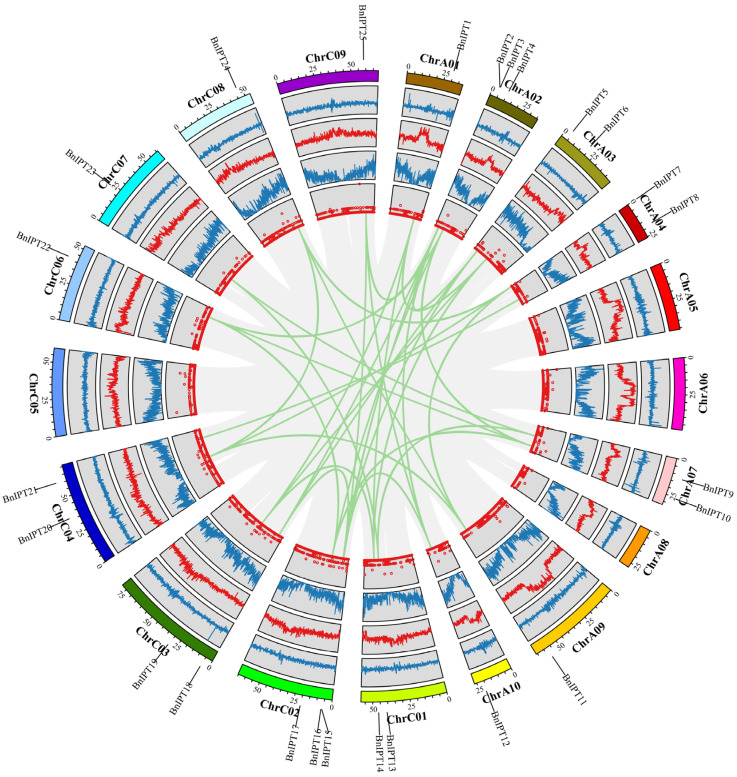
Collinearity of *BnIPT* genes. The circles in the figure from inside to outside represent the unknown base N ratio, gene density, GC ratio, GC skew, and chromosome length of the *B. napus* genome.

To investigate the covariance of *IPTs, BnIPT26*, which was localized on pseudochromosomes, was removed, and maps of collinearity were drawn for oilseed rape (*B. napus*), cabbage (*B. rapa*), and kale (*B. oleracea*) (Figure 4). It was found that all 25 *BnIPTs* were collinear with the *IPT* genes of *B. rapa* and *B. oleracea*, and 33 pairs of collinear genes existed between *B. napus* and *B. rapa IPT* genes, while 31 pairs of collinear genes existed between *B. napus* and *B. oleracea IPT* genes (Table S2). *BnIPT15* and *BnIPT23* had the most collinear relationship with *B. rapa* and *B. oleracea IPT* genes (four pairs). Collinearity analysis of *BnIPTs* within the *B. napus* genome showed that there were 34 pairs of collinear genes among *BnIPTs*, among which *BnIPT18* had the most collinear relationships with other *BnIPTs*, having 4 pairs (Figure 5, Table S3). In order to understand the protein-coding sequence relationships of *BnIPT* collinear gene pairs, the KaKs value was calculated to understand their selection pressure relationships (Table S4). The results showed that all the Ka/Ks values of these 34 collinear gene pairs were less than 1, indicating that they were all subject to purifying selection.

### 2.6. Protein–Protein Interaction Networks Analysis and GO Enrichment

**Figure 6:**
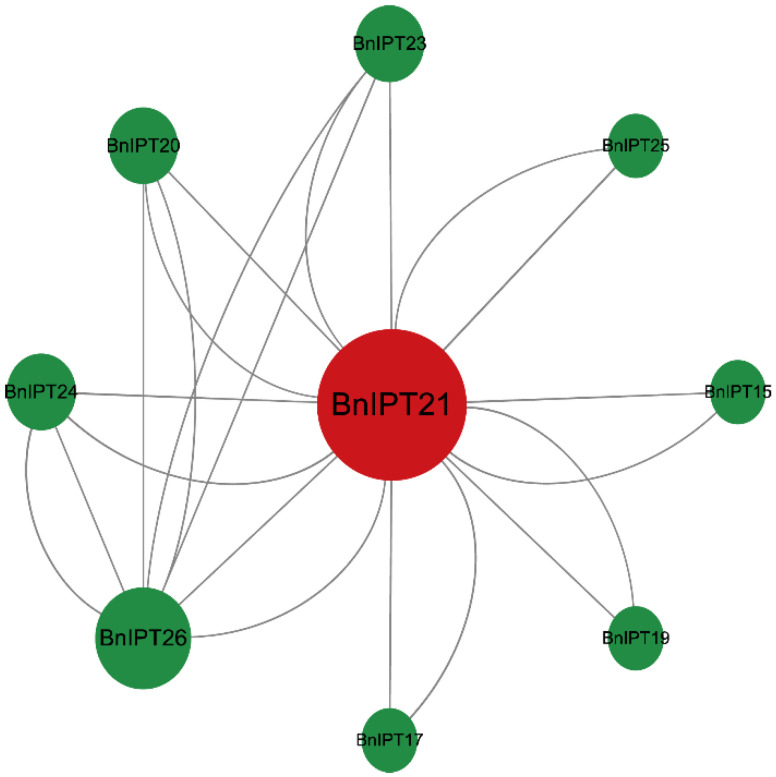
BnIPT proteins interaction network analysis.

**Figure 7:**
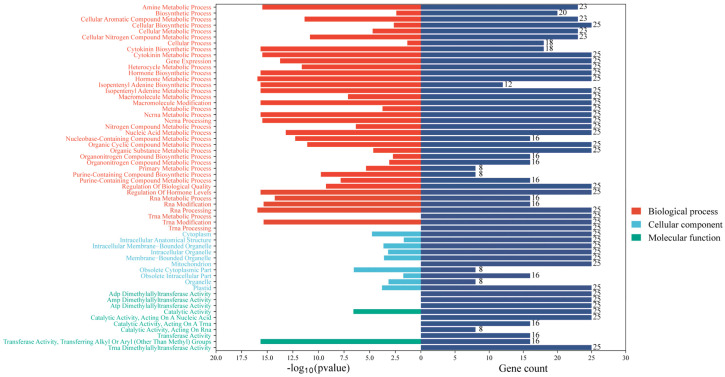
GO enrichment analysis of *BnIPT* genes.

PPI analysis showed that a total of BnIPT21, BnIPT26, BnIPT24, BnIPT20, BnIPT23, BnIPT25, BnIPT15, BnIPT19, and BnIPT17 were involved in the interactions, and there were 22 interactions (Figure 6). The larger the protein circle, the more proteins it might interact with. GO enrichment analysis showed that the Bnipt gene was associated with 38 biological processes, 10 molecular functions, and 10 cellular compositions (Figure 7). It is worth mentioning that *BnIPTs* were found to be associated with cellular nitrogen compound metabolic processes and nitrogen compound metabolic processes in addition to CK anabolism. Therefore, it was speculated that *BnIPTs* may have a role in the nitrogen metabolism pathway of oilseed rape.

### 2.7. BnIPT Transcriptome Expression Pattern Analysis

**Figure 8:**
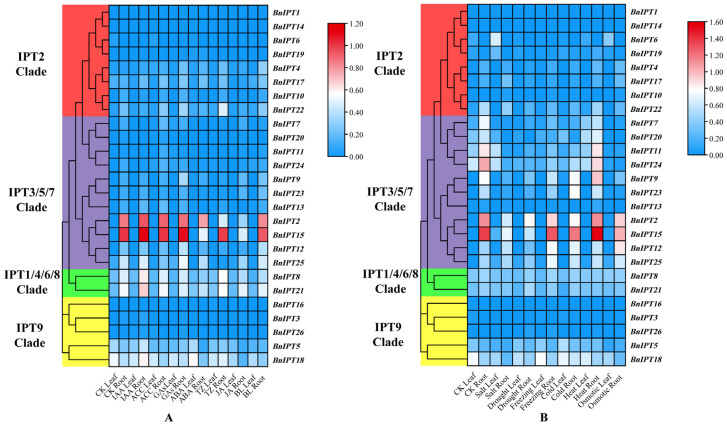
Analysis of expression patterns of *BnIPTs* under exogenous hormone and abiotic stress treatments. (**A**) The expression levels of *BnIPTs* in leaf and root under CK, IAA, ACC, GAs, ABA, TZ, JA, and BL treatments. (**B**) The expression levels of *BnIPTs* in leaf and root under CK, salt, drought, freezing, cold, heat, and osmotic treatments.

To investigate the gene expression pattern of the *BnIPTs* under exogenous hormone and abiotic stress treatments, the *BnIPTs* gene IDs were uploaded to the BNTIR website to obtain its expression patterns under control (CK), 10 µmol-L^−1^ growth hormone (IAA), ethylene (ACC), gibberellin (GAs), abscisic acid (ABA), trans-zeatin (TZ), jasmonic acid (JA), and oleuropein lactone (BL) treatment for 6 h and under control (CK), salt (200 mmol·L^−1^ NaCl treatment), drought (exposure to airflow for 1 h), freezing (stress at −4 °C for 3 h followed by recovery to 25 °C), cold (4 °C), heat (stress at 38 °C for 3 h followed by recovery to 25 °C), and osmosis (300 mmol·L^−1^ mannitol) treatments for 24 h. Log_10_(TPM + 1) was calculated to show the amount of gene expression, and then the data were visualized using the TBTools software. For exogenous hormone treatments, most genes were not expressed or expressed at low levels in leaves and roots under different treatments; however, in roots, *BnIPT2* was highly expressed under CK, IAA, ACC, GAs, ABA, and BL treatments; BnIPT15 was highly expressed under CK, IAA, ACC, GAs, TZ, and BL treatments; *BnIPT8*, *BnIPT8*, and *BnIPT21* were highly expressed under IAA treatment (Figure 8A). For abiotic stress treatments, most of the genes were equally absent or expressed at low levels in leaves and roots under different treatments; however, in roots, *BnIPT2* and *BnIPT15* were highly expressed under CK, freezing, heat, and osmotic stress treatments, and *BnIPT15* were highly expressed under cold stress. *BnIPT11* and *BnIPT24* were highly expressed under CK and heat stress; BnIPT11 and BnIPT24 were highly expressed under CK and heat stress; and *BnIPT9* was highly expressed under heat stress. In general, some *BnIPTs* showed high expression in roots under exogenous hormones or adversity stress, reflecting their role in regulating the growth and development and stress tolerance of rapeseed (Figure 8B).

### 2.8. Expression Analysis of BnIPTs in Response to Lack of Nitrogen

**Figure 9:**
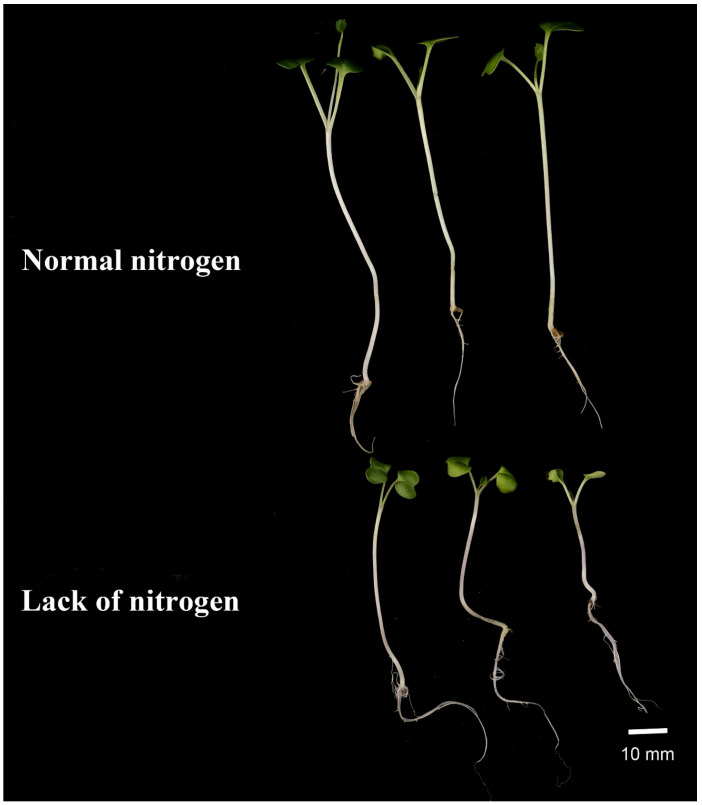
Growth in seedling stage of *B. napus* under two nitrogen levels.

**Figure 10:**
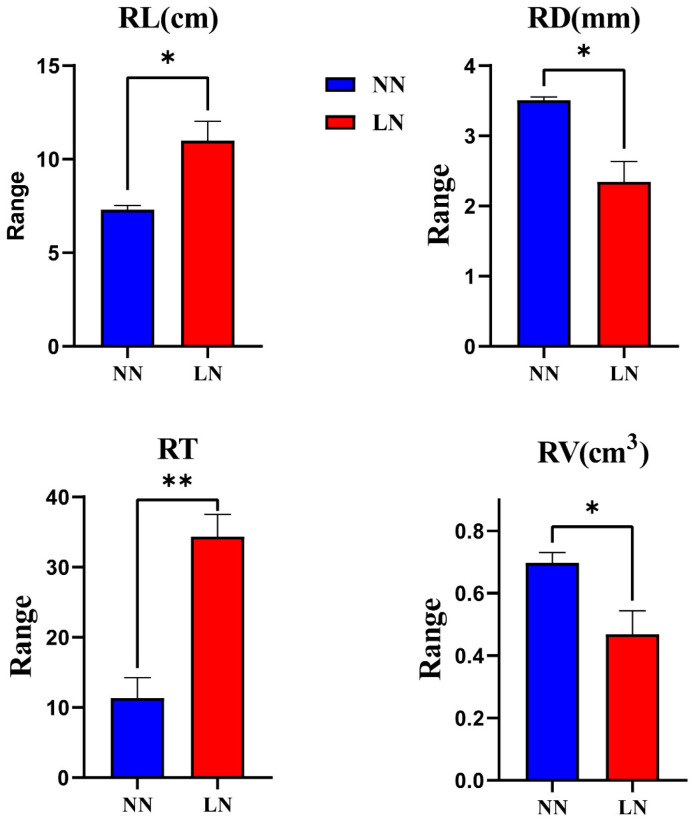
Root morphological indexes under two nitrogen levels in seedling stage of *B. napus.* RL: total root length, RD: decreased the mean root diameter, RT: the number of root tips, and RV: root volume. *: significant differences between treatments at *p* ≤ 0.05. **: significant differences between treatments at *p* ≤ 0.01.

CKs are involved in the regulation of nitrogen metabolism in plants, and *IPTs* are able to respond to nitrogen signals in plants. Therefore, to investigate the effect of *BnIPTs* on the response of oilseed rape to lack of nitrogen stress conditions, two hydroponic environments, normal nitrogen (NN, N: 6 mmol·L^−1^) and lack of nitrogen (LN, N: 0), were set up to cultivate oilseed rape, and root morphology data were obtained by WinRHIZO software. The expression of some *BnIPTs*, which were presumed to be involved in the regulation of abiotic stress tolerance in rapeseed, was examined by the qPCR method. The results showed that LN significantly inhibited the upper ground growth of rapeseed, but it was observed that rapeseed would respond to the unfavorable conditions caused by LN conditions by changing the root morphology (Figure 9). Analysis of the root morphology of rapeseed under both N levels showed that LN conditions increased the total root length (RL) and the number of root tips (RTs) and decreased the mean root diameter (RD) and root volume (RV) of rapeseed roots compared to NN conditions (Figure 10). This showed that LN conditions also inhibited the growth of rapeseed roots, causing its RD and RV to decrease, but it captured more N nutrients from the outside through root elongation and an increase in the number of root tips to reduce the inhibitory effect of LN conditions on its growth.

**Figure 11:**
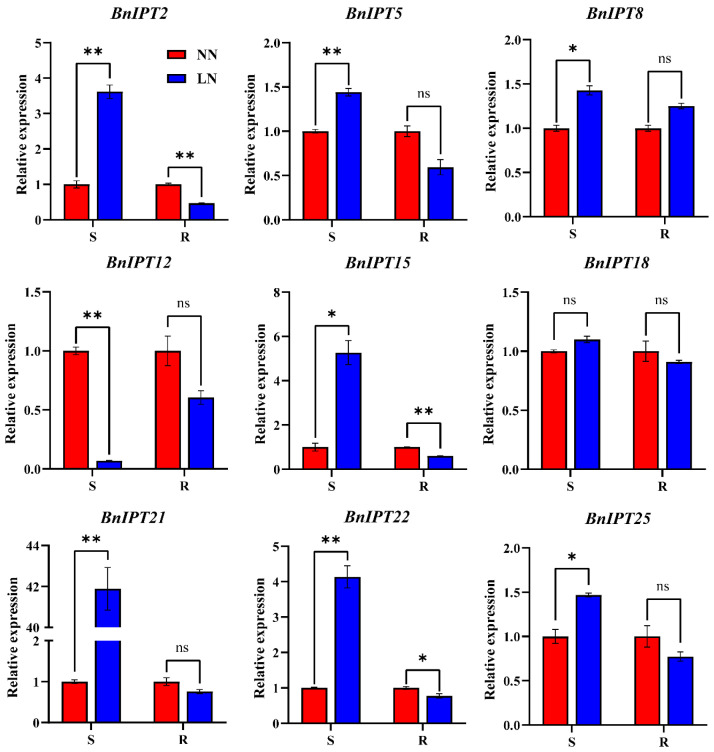
The relative expression of *BnIPT2*, *BnIPT5*, *BnIPT8*, *BnIPT12*, *BnIPT15*, *BnIPT18*, *BnIPT21*, *BnIPT22,* and *BnIPT25* in shoots (S) and roots (R) under normal nitrogen (NN) and lack of nitrogen (LN) conditions. ns: no significant differences between treatments. *: significant differences between treatments at *p* ≤ 0.05. **: significant differences between treatments at *p* ≤ 0.01.

For the S, compared with NN conditions, LN conditions caused significant up-regulation of *BnIPT2*, *BnIPT5*, *BnIPT8*, *BnIPT15*, *BnIPT21*, *BnIPT22*, and *BnIPT25*, especially of *BnIPT21*, which was up-regulated more than 40-fold (Figure 11); *BnIPT18* also showed an up-regulation trend, but the difference was not significant; on the contrary, *BnIPT12* was significantly down-regulated. For the R, *BnIPT2*, *BnIPT15*, and *BnIPT22* were significantly down-regulated; *BnIPT5*, *BnIPT12*, *BnIPT18*, *BnIPT21*, and *BnIPT25* showed a down-regulation trend, but the difference was not significant; and *BnIPT8* showed an up-regulation trend, but the difference was not significant. Overall, *BnIPTs* showed a trend of up-regulation in S and down-regulation in R in response to the nitrogen deficiency signal, indicating that they may affect the process of nitrogen transport and redistribution to enhance the resistance of oilseed rape in response to LN conditions.

## 3. Materials and Methods

### 3.1. Identification of BnIPT Family Members and Chromosome Mapping

The sequence files of ZS11 (a variety of *B.napus*), cabbage (*Brassica rapa*), and kale (*Brassica oleracea*) in kale-type oilseed rape (*B. napus*) were obtained from the BRAD database [27]. A total of 9 AtIPT protein sequences were downloaded from the Arabidopsis genome database (TAIR: https://www.arabidopsis.org/, accessed on 22 July 2022) as seed sequences, and BLASTp was used to search for possible BnIPT proteins (e Value < 1 × 10^−10^) in the whole protein sequence of *B. napus*. The IPPT conserved domain (PF01715) file download from the Pfam database [28] was used to search the possible BnIPT proteins too by using the HMMER software (http://www.hmmer.org/, accessed on 22 July 2022), combining the BLAST results to obtain the BnIPT hypothetical proteins. The hypothetical protein sequences were uploaded to the NCBI-CDD website (https://www.ncbi.nlm.nih.gov/cdd/, accessed on 22 July 2022) for further confirmation, and 26 members of the *BnIPT* gene family were identified finally. The nomenclature of the *BnIPTs* was determined based on their orders in chromosomal positions, and they were named *BnIPT1*~*BnIPT26*. The physicochemical properties of the BnIPT proteins were predicted using the Protparam function in the ExPASy website [29], and the subcellular localization prediction results were implemented by Plant-mPLoc [30]. The secondary structures of the BnIPT proteins were predicted using the SMOPA online tool, and the protein tertiary structures were predicted by SWISS-MODEL based on homology modeling [31,32]. The chromosomal location information of the *BnIPTs* was retrieved using the downloaded annotation files and visualized by TBTools [33].

### 3.2. Phylogenetic Analysis

The IPT protein sequences of *A. thaliana* and rice (*Oryza sativa*) were downloaded from TAIR and the Phytome database (https://phytozome-next.jgi.doe.gov/, accessed on 22 July 2022). Multiple sequence alignment by ClustalW, the phylogenetic tree was obtained by adjusting the parameters to neighbor-joining (NJ) and 1000 bootstrap replicates in MEGA 11 software [34], and the phylogenetic tree was embellished in the online website iTOL [35].

### 3.3. Prediction of Gene Structures, Protein Conserved Motifs, and Promoter Cis-Acting Elements

CDS/UTR region information and gene structure information of BnIPT gene family members were obtained from the NCBI-CDD website and the Pfam database. The conserved motifs of BnIPT proteins were obtained by using MEME 5.5.1 website [36], and the cis-acting elements of the upstream 2000 bp of the promoter region of *BnIPTs* were predicted by the PlantCARE website [37]. All data information visualization was performed using TBTools.

### 3.4. IPT Genes Collinearity Analysis

Analysis of the collinear relationship of IPT genes between the genomes of *B. napus*, *B. rapa*, and *B. oleracea*, as well as within the genomes of *B. napus*, was finished by using MCScanX software [38]. Collinearity information visualization was performed using TBTools. Calculation of the Ka/Ks value was realized by using KaKs_calculator 3.0 software [39].

### 3.5. Protein–Protein Interaction Networks and GO Enrichment Analysis of BnIPTs

First, the BnIPT protein sequences were uploaded to the STRING database [40] to obtain their protein–protein interaction (PPI) network information and then visualized by Cytoscape software [41]. To further investigate the functions of *BnIPTs*, they were subjected to gene ontology (GO) functional annotation and classification. The whole protein sequences of ZS11 were aligned online by the eggNOG-mapper database, and the annotation information of the whole proteins of ZS11 was obtained [42].

### 3.6. Transcriptome Expression Pattern Analysis

Data of expression levels displayed by TPM (transcripts per million reads) of *BnIPTs* in leaves and roots of ZS11 under different abiotic stress and different exogenous hormone treatments were obtained from the BnTIR database [43], and expression heat maps were produced using TBTools software.

### 3.7. Plants Materials and Treatment

ZS11 was selected as the experiment material. After germination of materials in 1/8 Hoagland solution for 7 d, 10 healthy and uniformly growing seedlings were taken, and 5 plants each were placed in normal N (NN: 6 mol-L-1) and lack of nitrogen (LN: 0) culture solutions (Table 2). The N concentrations of both nutrient solutions were adjusted by adjusting the concentrations of KNO_3_ and Ca(NO_3_)_2_·4H_2_O. The concentrations of KCl and CaCl_2_ were adjusted to maintain the concentrations of K^+^ and Ca^2+^. After 7 d of incubation in each treatment, the root morphological data were obtained by WinRHIZO software, and the above-ground (S) and the below-ground roots (R) were harvested and stored in liquid nitrogen, respectively. The experiment was carried out at the Genome Center of the Straits Joint Research Institute of Fujian Agriculture and Forestry University (26°11 N, 119°23 E).

### 3.8. Relative Expression of BnIPT Genes under Different Nitrogen Levels

Total RNA was extracted from S and R of ZS11 in NN and LN treatments using an Eastep^®^ Super Total RNA Extraction Kit (Promega, Shanghai, China), with RNA concentrations and absorbancy determined using a NanoDrop One spectrophotometer (Thermo Fisher Scientific, Waltham, USA). OD260/OD280 values of total RNA were in the range of 1.8–2.0. cDNA was obtained by reverse transcription of total RNA using a TransScipt^®^ One-Step gDNA Removal and cDNA Synthesis SuperMix (TransScript, Beijing, China). Fluorescent quantification primers were designed to amplify fragments of 50–300 bp in length using the BnIPTs CDS region sequences as templates (Table 3) for quantitative real-time PCR (qPCR) experiments by Primer Premier 5.0 software. qPCR analyses were performed on a qTOWER^3^ G (analytik jena, Jena, Germany). All reactions included 10.0 μL of PerfectStrat^®^ 2 × Green qPCR SuperMix (TransScript, Beijing, China), 2 μL of cDNA template, 0.4 μL of forward and reverse primers, and 7.2 μL of ddH_2_O, made up to a 20 μL volume. The reaction procedure consisted of 94 °C for 30 s, followed by 40 cycles of 94 °C for 5 s, followed by 40 cycles of 60 °C for 30 s to construct a melting curve. Three biological replicates were designed for each group of experiments, and the 2^−ΔΔCT^ method was used to calculate the relative expression of *BnIPTs*. *BnActin7* was selected as the internal reference gene, which was reported to be validated by the expression of the reference gene of *B. napus* under low nitrogen stress [44]. 

### 3.9. Statistical Analysis

The data were analyzed by SPSS version 22.0 software. The tables were carried out with Microsoft Excel version 2021, and the figures of statistical analysis and qPCR were completed by Origin Lab version 2023 software and GraphPad Prism version 8.0 software.

## 4. Discussion

Gene family analysis can provide an effective method to better understand gene structure, function, and evolution. *IPTs* are key genes that regulate the synthesis of CKs and play an important role in the regulation of CK levels in plants. Previous work identified 19 *BnIPTs* in *B. napus* by RNA-seq data [45]. The predecessors were limited by immature sequencing technology, unable to obtain high-quality *B. napus* genome data, and thus, could not mine all potential *BnIPT* gene family members of the *B. napus* genome by the sequencing methods available at the time. With the continuous development of sequencing technology and bioinformatics tools, high-quality genome-wide information of various representative varieties of *B. napus*, such as *Darmor-bzh* and ZS11, was published [46,47]. In this study, the AtIPT protein sequences were used as the seed sequence and combined with the results of a pfam structural domain search; 26 members of the *BnIPT* gene family were finally identified, distributed on 15 identified chromosomes and 1 pseudochromosome. Based on the phylogenetic analysis of protein sequences from three species, Arabidopsis, rice, and oilseed rape, *BnIPTs* were asymmetrically distributed among four clades. Analysis of the number of BnIPT proteins in each clade revealed that the IPT2 Clade had the lowest percentage of BnIPT proteins, 28.6%, while the remaining three clades had more than 50% (Figure 2). It suggested that the oilseed rape IPT2 Clade was more conserved in terms of gene evolution compared to Arabidopsis and rice, while gene family duplication events may have occurred at the genome-wide level in oilseed rape on the other three clades. Analysis of the gene structures and conserved structural domains of *BnIPT* showed that these genes are evolutionarily conserved across species, and this conservation is evident in including Arabidopsis [48], rice [49], and apples (*Malus pumila*) [50]. By analyzing the collinear data of the *IPT* gene family among the genomes of oilseed rape, cabbage, and kale, 33 pairs of collinear genes in oilseed rape and cabbage and 31 pairs of collinear genes in oilseed rape and kale were found; meanwhile, 34 pairs of collinear genes existed in *B. napus*, and their Ka/Ks values were all less than 1. The number of collinear genes among species of the *IPT* gene family was larger than the number of existing *BnIPTs*, indicating that *IPT* genes were related in evolution and had evolutionary conservation among species and within species.

Different biological functions can be produced by the interaction between proteins [51]. In this study, 22 interactions were found between 9 proteins: BnIPT21, BnIPT26, BnIPT24, BnIPT20, BnIPT23, BnIPT25, BnIPT15, BnIPT19, and BnIPT17. The GO enrichment analysis revealed that *BnIPTs* were involved in the cytokinin biosynthetic process, cytokinin metabolic process, isopentenyl adenine biosynthetic process, and isopentenyl adenine metabolic process, further verifying that *BnIPTs* played an important role in the synthesis and metabolism of CKs. The role of *IPT* genes in the anabolic metabolism of CKs has been reported in many plants. For example, Li et al. [52] added thiadiazole (TDZ, CKs artificial plant growth regulator) to phalaenopsis and found that the in vitro bud proliferation would be promoted with the increase in TDZ concentration, and *PaIPT1* was highly expressed by TDZ treatment; the levels of CKs of *PaIPT1* overexpressed plants would be increased compared to normal plants. For apples, the content of endogenous CKs of *MdIPT8* overexpressed plants could be increased in response to anthracnose infection, and the application of exogenous CKs would enhance the resistance of apples to anthracnose [53].

*IPTs* are involved in processes such as plant growth and development and response to abiotic stress, but their expression patterns vary among species [54]. Transcriptome data showed that the S and R of *BnIPTs* exhibited different expression patterns under different exogenous hormone treatments and stress treatment conditions. Under most conditions, *BnIPTs* were not expressed significantly. However, *BnIPT2* and *BnIPT15* showed high expression in R under different treatments; *BnIPT9*, *BnIPT11*, and *BnIPT24* were also expressed in R under heat stress. It suggested that oilseed rape may be able to maintain its growth by altering expression levels of *BnIPTs* in R in response to adverse external environments. Most of the *IPT* mutants of Arabidopsis have confirmed the existence of an inhibitory effect of CKs on root growth [55]; however, overexpression of *IPT* in plants could promote the translocation of CKs bottom-up and delay leaf senescence: overexpression of tomato *IPTs* under a root-specific promoter resulted in a slight increase the concentration of endogenous CKs in the plant, but the proportion of CKs derivatives in the root was significantly altered, resulting in biosynthesis and metabolism to be altered; while leaf senescence of tomatoes was regulated by the CK-mediated root–shoot pathway, and CK-mediated signals were transferred from xylem to leaves, thus altering the level of CKs biosynthesis in leaves and delaying leaf senescence [56].

**Figure 12:**
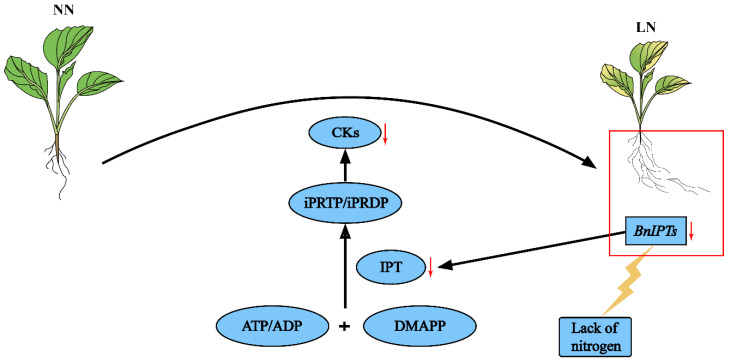
Mechanism of *BnIPTs* on root growth of rape.

Based on the results of GO enrichment analysis, it was found that the *BnIPTs* may also be associated with cellular nitrogen compound metabolic processes, nitrogen compound metabolic processes, organic nitrogen compound biosynthetic processes, and organic nitrogen compound metabolic processes. Therefore, it was speculated that *BnIPTs* might be involved in regulating the process of oilseed rape in response to nitrogen deficiency stress. Previous studies have shown that high concentrations of CKs could regulate shoot apical meristem development to promote apical dominance as well as inhibit adventitious root development [57]. In our research, compared with the NN conditions, the most *BnIPTs* showed up-regulated expression in S and down-regulated expression in R under LN conditions (Figure 11), indicating that *BnIPTs* may regulate the increase in CK concentration in S and the decrease in CK concentration in R, especially by changing the root morphological index (Figure 10), in order to cope with the adverse effects of LN conditions. Under LN conditions, the down-regulation of the *BnIPTs* in R inhibited the synthesis process of IPT proteins so that the synthesis of iPRP/iPDP (isopentenyl adenosine-5-phosphate/isopentenyl adenosine-5-diphosphate) from ATP/ADP (adenosine nucleoside triphosphate/adenosine diphosphate) and DMAAP (dimethylene propyl pyrophosphate) participated by the BnIPT proteins was hindered, which in turn inhibited the subsequent synthesis of CKs with a series of reactions, making the decrease in the concentration of CKs in R, prompting it to become elongated and increase the number of root tips to enhance its ability to absorb external nitrogen nutrients from the outside (Figure 12). However, *BnIPT12* showed a down-regulation trend in both S and R in response to LN stress, and the S was significantly down-regulated, while nine of the other genes were collinear with it and showed such an expression pattern, suggesting that there may be some mechanism (e.g., epistatic modification, etc.) leading to the diverse expression of collinear genes [58].

## 5. Conclusions

The genome of *B. napus* was identified to contain 26 *BnIPTs* distributed on 15 identified chromosomes and 1 pseudochromosome. Their phylogenetic relationships, protein physicochemical properties and structure, subcellular localization, gene structure, promoter cis-acting elements, covariance relationships, protein interaction networks, and GO enrichment were predicted and analyzed, and their expression patterns at the transcriptional level under exogenous hormone and abiotic stress treatments and differences in expression under NN and LN conditions were investigated. The results showed that the expression patterns of *BnIPTs* under exogenous hormone and abiotic stress treatments were different; under LN conditions, the growth and development of rapeseed could be maintained by increasing the expression of *BnIPTs* in S and inhibiting the expression of *BnIPTs* in R.

## Figures and Tables

**Table 1:** Predicted secondary structure of BnIPT proteins.

Protein Name	Hh	Ee	Cc	Protein Name	Hh	Ee	Cc
BnIPT1	45.06%	12.04%	42.90%	BnIPT14	45.68%	11.42%	42.90%
BnIPT2	46.83%	11.78%	41.39%	BnIPT15	49.40%	11.98%	38.62%
BnIPT3	47.00%	13.43%	39.57%	BnIPT16	51.45%	8.44%	40.11%
BnIPT4	46.55%	11.21%	42.24%	BnIPT17	42.82%	13.79%	43.39%
BnIPT5	48.63%	10.22%	41.15%	BnIPT18	45.36%	13.61%	41.04%
BnIPT6	45.40%	11.96%	42.64%	BnIPT19	45.85%	12.31%	41.85%
BnIPT7	51.50%	11.08%	37.43%	BnIPT20	49.40%	11.98%	38.62%
BnIPT8	51.39%	10.13%	38.48%	BnIPT21	52.80%	8.84%	38.36%
BnIPT9	47.59%	12.65%	39.76%	BnIPT22	45.48%	11.45%	43.07%
BnIPT10	46.85%	11.71%	41.44%	BnIPT23	46.69%	12.35%	40.96%
BnIPT11	49.10%	10.54%	40.36%	BnIPT24	51.51%	10.24%	38.25%
BnIPT12	50.60%	11.38%	38.02%	BnIPT25	48.80%	11.08%	40.12%
BnIPT13	51.10%	12.23%	36.68%	BnIPT26	44.61%	12.07%	43.32%

**Table 2:** Formulation of normal and lack of nitrogen nutrient solutions.

Chemical Reagent	Mother Liquor (g/L)	Normal Nitrogen Nutrient Solution (mL/L) ^1^	Lack of Nitrogen Nutrition Solution (mL/L)
KNO_3_	102.00	5.00	0
MgSO_4_·7H_2_O	98.00	5.00	5.00
KH_2_PO_4_	28.00	5.00	5.00
Ca(NO_3_)_2_·4H_2_O	236.00	0.50	0
FeSO_4_·7H_2_O	2.80	5.00	5.00
EDTA-2Na	3.72	5.00	5.00
MnCl_2_·4H_2_O	3.62	0.25	0.25
ZnSO_4_·7H_2_O	0.44	0.25	0.25
CuSO_4_·5H_2_O	0.16	0.25	0.25
H_3_BO_3_	5.72	0.25	0.25
Na_2_MoO_4_·4H_2_O	0.18	0.25	0.25
KCl	75.50	0	5.00
CaCl_2_	111.00	4.50	5.00

1: Adding mother liquor volume per liter of nutrient solution.

**Table 3:** qPCR primer sequence of *BnIPT* genes.

Gene Name	Forward Primer (5′→3′)	Reverse Primer (3′→5′)	Amplicon Length (bp)
*BnIPT2*	CCGACAAGATCCAAGTCTACAA	AAATCCTCGTGAGTGTCTTCC	118
*BnIPT5*	AGGCCTTGATGTTGGATCAG	CCTTGAGAGGGATGCAGAATAT	90
*BnIPT8*	GCGAAGGTGGTCGTGATAAT	TTGCGTCGGCGTTTATGA	103
*BnIPT9*	CTCAACACTCTCGGCTAACAA	AGGAAACGTCGACCCAAATAA	137
*BnIPT12*	GTCTAGACATCGTCACCAACAA	TAATCCTCCGCCGTGAAATC	109
*BnIPT15*	GGAGAACACTCACGACGATTT	AGAATTAGAACCACCGGCTATG	115
*BnIPT18*	GGTGACTAGCAGTAGCGTATTC	TCGAAGTGTTCCCGTTTAGAG	131
*BnIPT21*	CGGAATCGAGGAAGAGAAGATG	GAAGTGAGACGCCAAGTCTAC	106
*BnIPT22*	CCGTCTTTCTCCTCACATTCTC	GCTCCTAAGATGACGACGATTT	101
*BnIPT25*	ACAAAGTCACTCCAGAGGAAAG	AGTGCTTCACGCTGGTAATC	106
*BnActin7*	TGGGTTTGCTGGTGACGAT	TGCCTAGGACGACCAACAATACT	63

## Data Availability

All data analyzed during this study are included in this article and its additional files.

## Supplementary Materials

The following supporting information can be downloaded at: https://www.mdpi.com/article/10.3390/plants12112166/s1, Table S1: Prediction of physicochemical properties and subcellular localization of BnIPT proteins; Table S2: Collinear gene pairs of *IPT* genes in *B. napus*, *B. rapa* and *B. oleracea*; Table S3: Collinear gene pairs of *IPT* genes in *B. napu*; Table S4: Ka/Ks calculation of *BnIPTs*; Figure S1: Tertiary structure prediction of BnIPT proteins.

## References

[1] Waadt R. (2020). Phytohormone signaling mechanisms and genetic methods for their modulation and detection. Curr. Opin. Plant. Biol..

[2] Kieber J.J., Schaller G.E. (2018). Cytokinin signaling in plant development. Development.

[3] Cortleven A., Leuendorf J.E., Frank M., Pezzetta D., Bolt S., Schmulling T. (2019). Cytokinin action in response to abiotic and biotic stresses in plants. Plant Cell Environ..

[4] Mik V., Szucova L., Spichal L., Plihal O., Nisler J., Zahajska L., Dolezal K., Strnad M. (2011). N9-Substituted N(6)-[(3-methylbut-2-en-1-yl)amino]purine derivatives and their biological activity in selected cytokinin bioassays. Bioorg. Med. Chem..

[5] Wybouw B., De Rybel B. (2019). Cytokinin—A Developing Story. Trends Plant Sci..

[6] Golovko A., Sitbon F., Tillberg E., Nicander B. (2002). Identification of a tRNA isopentenyltransferase gene from *Arabidopsis thaliana*. Plant Mol. Biol..

[7] Gajdosova S., Spichal L., Kaminek M., Hoyerova K., Novak O., Dobrev P.I., Galuszka P., Klima P., Gaudinova A., Zizkova E. (2011). Distribution, biological activities, metabolism, and the conceivable function of cis-zeatin-type cytokinins in plants. J. Exp. Bot..

[8] Hnatuszko-Konka K., Gerszberg A., Weremczuk-Jezyna I., Grzegorczyk-Karolak I. (2021). Cytokinin Signaling and De Novo Shoot Organogenesis. Genes.

[9] Takei K., Sakakibara H., Sugiyama T. (2001). Identification of genes encoding adenylate isopentenyltransferase, a cytokinin biosynthesis enzyme, in *Arabidopsis thaliana*. J. Biol. Chem..

[10] Kakimoto T. (2001). Identification of plant cytokinin biosynthetic enzymes as dimethylallyl diphosphate: ATP/ADP isopentenyltransferases. Plant Cell Physiol..

[11] Zhu Y., Jiang X., Zhang J., He Y., Zhu X., Zhou X., Gong H., Yin J., Liu Y. (2020). Silicon confers cucumber resistance to salinity stress through regulation of proline and cytokinins. Plant Physiol. Biochem..

[12] Beznec A., Faccio P., Miralles D.J., Abeledo L.G., Oneto C.D., Garibotto M.B., Gonzalez G., Moreyra F., Elizondo M., Ruiz M. (2021). Stress-induced expression of IPT gene in transgenic wheat reduces grain yield penalty under drought. J. Genet. Eng. Biotechnol..

[13] Xu Y., Gianfagna T., Huang B. (2010). Proteomic changes associated with expression of a gene (ipt) controlling cytokinin synthesis for improving heat tolerance in a perennial grass species. J. Exp. Bot..

[14] Prerostova S., Cerny M., Dobrev P.I., Motyka V., Hluskova L., Zupkova B., Gaudinova A., Knirsch V., Janda T., Brzobohaty B. (2020). Light Regulates the Cytokinin-Dependent Cold Stress Responses in Arabidopsis. Front. Plant Sci..

[15] Sakakibara H. (2021). Cytokinin biosynthesis and transport for systemic nitrogen signaling. Plant J..

[16] Zhang W.Y., Zhou Y.J., Li C.Q., Zhu K.Y., Xu Y.J., Wang W.L., Liu L.J., Zhang H., Gu J.F., Wang Z.Q. (2022). Post-anthesis moderate soil-drying facilitates source-to-sink remobilization of nitrogen via redistributing cytokinins in rice. Field Crops Res..

[17] Shibasaki K., Takebayashi A., Makita N., Kojima M., Takebayashi Y., Kawai M., Hachiya T., Sakakibara H. (2021). Nitrogen Nutrition Promotes Rhizome Bud Outgrowth via Regulation of Cytokinin Biosynthesis Genes and an *Oryza longistaminata* Ortholog of FINE CULM 1. Front. Plant Sci..

[18] Beszterda M., Nogala-Kałucka M. (2019). Current Research Developments on the Processing and Improvement of the Nutritional Quality of Rapeseed (*Brassica napus* L.). Eur. J. Lipid Sci. Technol..

[19] Moggré G.-J., Alayon Marichal M., Sowersby T., Grosvenor A., Gathercole J., Moreno T. (2021). Protein Recovery from New Zealand Oil Rapeseed (*Brassica napus*) Cake. Waste Biomass Valorization.

[20] Li J., Han Z., Xian M. (2022). Exploration and application of agriculture-tourism technologies based on rape flowers in rural revitalization of China. Oil Crop Sci..

[21] Ahmed M., Rauf M., Akhtar M., Mukhtar Z., Saeed N.A. (2020). Hazards of nitrogen fertilizers and ways to reduce nitrate accumulation in crop plants. Environ. Sci. Pollut. Res. Int..

[22] Rathke G., Behrens T., Diepenbrock W. (2006). Integrated nitrogen management strategies to improve seed yield, oil content and nitrogen efficiency of winter oilseed rape (*Brassica napus* L.): A review. Agric. Ecosyst. Environ..

[23] Sieling K., Kage H. (2010). Efficient N management using winter oilseed rape. A review. Agron. Sustain. Dev..

[24] Liu Q., Ren T., Zhang Y., Li X., Cong R., White P.J., Lu J. (2019). Yield loss of oilseed rape (*Brassica napus* L.) under nitrogen deficiency is associated with under-regulation of plant population density. Eur. J. Agron..

[25] Paul P., Aithal P., Ripu Ranjan Sinha R.R.S., Saavedra M.R., Aremu P.S.B. (2019). Agro Informatics with its Various Attributes and Emergence: Emphasizing Potentiality as a Specialization in Agricultural Sciences—A Policy Framework. IRA Int. J. Appl. Sci..

[26] Ton L.B., Neik T.X., Batley J.J.G. (2020). The use of genetic and gene technologies in shaping modern rapeseed cultivars (*Brassica napus* L.). Genes.

[27] Chen H., Wang T., He X., Cai X., Lin R., Liang J., Wu J., King G., Wang X. (2022). BRAD V3.0: An upgraded Brassicaceae database. Nucleic Acids Res..

[28] Mistry J., Chuguransky S., Williams L., Qureshi M., Salazar G.A., Sonnhammer E.L.L., Tosatto S.C.E., Paladin L., Raj S., Richardson L.J. (2021). Pfam: The protein families database in 2021. Nucleic Acids Res..

[29] Duvaud S., Gabella C., Lisacek F., Stockinger H., Ioannidis V., Durinx C. (2021). Expasy, the Swiss Bioinformatics Resource Portal, as designed by its users. Nucleic Acids Res..

[30] Chou K.C., Shen H.B. (2010). Plant-mPLoc: A top-down strategy to augment the power for predicting plant protein subcellular localization. PLoS ONE.

[31] Combet C., Blanchet C., Geourjon C., Deleage G. (2000). NPS@: Network Protein Sequence Analysis. Trends Biochem. Sci..

[32] Waterhouse A., Bertoni M., Bienert S., Studer G., Tauriello G., Gumienny R., Heer F.T., de Beer T.A.P., Rempfer C., Bordoli L. (2018). SWISS-MODEL: Homology modelling of protein structures and complexes. Nucleic Acids Res..

[33] Chen C., Chen H., Zhang Y., Thomas H.R., Frank M.H., He Y., Xia R. (2020). TBtools: An Integrative Toolkit Developed for Interactive Analyses of Big Biological Data. Mol. Plant.

[34] Tamura K., Stecher G., Kumar S. (2021). MEGA11: Molecular Evolutionary Genetics Analysis Version 11. Mol. Biol. Evol..

[35] Letunic I., Bork P. (2021). Interactive Tree Of Life (iTOL) v5: An online tool for phylogenetic tree display and annotation. Nucleic Acids Res..

[36] Nystrom S.L., McKay D.J. (2021). Memes: A motif analysis environment in R using tools from the MEME Suite. PLoS Comput. Biol..

[37] Lescot M., Dehais P., Thijs G., Marchal K., Moreau Y., Van de Peer Y., Rouze P., Rombauts S. (2002). PlantCARE, a database of plant cis-acting regulatory elements and a portal to tools for in silico analysis of promoter sequences. Nucleic Acids Res..

[38] Wang Y., Tang H., Debarry J.D., Tan X., Li J., Wang X., Lee T.H., Jin H., Marler B., Guo H. (2012). MCScanX: A toolkit for detection and evolutionary analysis of gene synteny and collinearity. Nucleic Acids Res..

[39] Zhang Z. (2022). KaKs_Calculator 3.0: Calculating Selective Pressure on Coding and Non-coding Sequences. Genom. Proteom. Bioinform..

[40] Szklarczyk D., Gable A.L., Nastou K.C., Lyon D., Kirsch R., Pyysalo S., Doncheva N.T., Legeay M., Fang T., Bork P. (2021). The STRING database in 2021: Customizable protein-protein networks, and functional characterization of user-uploaded gene/measurement sets. Nucleic Acids Res..

[41] Doncheva N.T., Morris J.H., Gorodkin J., Jensen L.J. (2019). Cytoscape StringApp: Network Analysis and Visualization of Proteomics Data. J. Proteome Res..

[42] Cantalapiedra C.P., Hernandez-Plaza A., Letunic I., Bork P., Huerta-Cepas J. (2021). eggNOG-mapper v2: Functional Annotation, Orthology Assignments, and Domain Prediction at the Metagenomic Scale. Mol. Biol. Evol..

[43] Liu D., Yu L., Wei L., Yu P., Wang J., Zhao H., Zhang Y., Zhang S., Yang Z., Chen G. (2021). BnTIR: An online transcriptome platform for exploring RNA-seq libraries for oil crop *Brassica napus*. Plant Biotechnol. J..

[44] Wang G.L., Ding G.D., Xu F.S., Cai H.M., Zou J., Ye X.S. (2014). Genotype differences in photosynthetic characteristics and nitrogen efficiency of new-type oilseed rape responding to low nitrogen stress. J. Agric. Sci..

[45] Song J., Jiang L., Jameson P.E. (2015). Expression patterns of *Brassica napus* genes implicate IPT, CKX, sucrose transporter, cell wall invertase, and amino acid permease gene family members in leaf, flower, silique, and seed development. J. Exp. Bot..

[46] Chalhoub B., Denoeud F., Liu S., Parkin I.A., Tang H., Wang X., Chiquet J., Belcram H., Tong C., Samans B. (2014). Plant genetics. Early allopolyploid evolution in the post-Neolithic *Brassica napus* oilseed genome. Science.

[47] Sun F., Fan G., Hu Q., Zhou Y., Guan M., Tong C., Li J., Du D., Qi C., Jiang L. (2017). The high-quality genome of *Brassica napus* cultivar ‘ZS11’ reveals the introgression history in semi-winter morphotype. Plant J..

[48] Le D.T., Nishiyama R., Watanabe Y., Vankova R., Tanaka M., Seki M., Ham L.H., Yamaguchi-Shinozaki K., Shinozaki K., Tran L.S. (2012). Identification and expression analysis of cytokinin metabolic genes in soybean under normal and drought conditions in relation to cytokinin levels. PLoS ONE.

[49] Ghosh A., Shah M.N.A., Jui Z.S., Saha S., Fariha K.A., Islam T. (2018). Evolutionary variation and expression profiling of Isopentenyl transferase gene family in *Arabidopsis thaliana* L. and *Oryza sativa* L.. Plant Gene.

[50] Tan M., Li G., Qi S., Liu X., Chen X., Ma J., Zhang D., Han M. (2018). Identification and expression analysis of the IPT and CKX gene families during axillary bud outgrowth in apple (*Malus domestica* Borkh.). Gene.

[51] Soleymani F., Paquet E., Viktor H., Michalowski W., Spinello D. (2022). Protein-protein interaction prediction with deep learning: A comprehensive review. Comput. Struct. Biotechnol. J..

[52] Li Y.Y., Hao Z.G., Miao S., Zhang X., Li J.Q., Guo S.X., Lee Y.I. (2022). Profiles of Cytokinins Metabolic Genes and Endogenous Cytokinins Dynamics during Shoot Multiplication In Vitro of Phalaenopsis. Int. J. Mol. Sci..

[53] Shi J., Zhang F., Su Y., Jiang Q., Yuan Y., Nie X., Zhou Y., Zhang X., Wang Z., Wang F. (2022). MdIPT8, an isopentenyl transferase enzyme, enhances the resistance of apple to *Colletotrichum gloeosporioides* infection. Sci. Hortic..

[54] Nguyen H.N., Lai N., Kisiala A.B., Emery R.J.N. (2021). Isopentenyltransferases as master regulators of crop performance: Their function, manipulation, and genetic potential for stress adaptation and yield improvement. Plant Biotechnol. J..

[55] Antoniadi I., Mateo-Bonmati E., Pernisova M., Brunoni F., Antoniadi M., Villalonga M.G., Ament A., Karady M., Turnbull C., Dolezal K. (2022). IPT9, a cis-zeatin cytokinin biosynthesis gene, promotes root growth. Front. Plant Sci..

[56] Glanz-Idan N., Lach M., Tarkowski P., Vrobel O., Wolf S. (2022). Delayed Leaf Senescence by Upregulation of Cytokinin Biosynthesis Specifically in Tomato Roots. Front. Plant Sci..

[57] Liu Y., Zhang M., Meng Z., Wang B., Chen M. (2020). Research Progress on the Roles of Cytokinin in Plant Response to Stress. Int. J. Mol. Sci..

[58] Fan X., Peng L., Zhang Y. (2022). Plant DNA Methylation Responds to Nutrient Stress. Genes.

