# Supplementary material for: Identification and Expression Analysis of the Isopentenyl Transferase (IPT) Gene Family under Lack of Nitrogen Stress in Oilseed (Brassica napus L.)
Source: Plants (Basel). 2023 May 30;12(11):2166. doi: 10.3390/plants12112166 (PMC10255845; doi:10.3390/plants12112166)
Supplement: Supplementary file 1 [file plants-12-02166-s001.zip › Table S1.pdf]

Table S1. Prediction of physicochemical properties and subcellular localization of BnIPT proteins.

Gene ID	Name	Number of Amino Acid	Mw (KDa)	PI	Gravy	Subcellular Localization
BnaA01G0322000ZS	*BnIPT1*	324	36.89	8.50	-0.335	Chloroplast
BnaA02G0076800ZS	*BnIPT2*	331	37.61	5.75	-0.223	Chloroplast
BnaA02G0083300ZS	*BnIPT3*	417	47.80	5.70	-0.474	Cell membrane/ chloroplast/ Mitochondria/ Nucleus
BnaA02G0174100ZS	*BnIPT4*	348	39.73	8.73	-0.422	Chloroplast/ Cytoplasm
BnaA03G0086000ZS	*BnIPT5*	401	46.18	6.14	-0.451	Chloroplasts/ Mitochondria
BnaA03G0357200ZS	*BnIPT6*	326	36.93	9.02	-0.303	Chloroplast
BnaA04G0002800ZS	*BnIPT7*	334	37.34	8.11	-0.137	Chloroplast
BnaA04G0179800ZS	*BnIPT8*	395	44.15	5.87	-0.375	Chloroplast/ Cytoplasm
BnaA07G0075700ZS	*BnIPT9*	332	37.15	8.68	-0.222	Chloroplast/ Cytoplasm/ Mitochondria
BnaA07G0275400ZS	*BnIPT10*	333	37.76	8.91	-0.335	Chloroplast
BnaA09G0568800ZS	*BnIPT11*	332	37.57	8.40	-0.187	Chloroplast
BnaA10G0183600ZS	*BnIPT12*	334	37.89	6.35	-0.236	Chloroplast
BnaC01G0355800ZS	*BnIPT13*	319	35.76	8.43	-0.254	Chloroplast
BnaC01G0398300ZS	*BnIPT14*	324	36.76	8.84	-0.327	Chloroplast
BnaC02G0090600ZS	*BnIPT15*	334	37.85	5.75	-0.197	Chloroplast/ Cytoplasm/ Mitochondria
BnaC02G0099100ZS	*BnIPT16*	379	43.49	5.40	-0.562	Chloroplast/ Cytoplasm/ Mitochondria
BnaC02G0224500ZS	*BnIPT17*	348	39.60	8.92	-0.407	Chloroplast/ Cytoplasm
BnaC03G0098500ZS	*BnIPT18*	463	52.23	7.03	-0.427	Chloroplast
BnaC03G0435200ZS	*BnIPT19*	325	37.06	8.87	-0.344	Chloroplast
BnaC04G0257300ZS	*BnIPT20*	334	37.60	6.96	-0.146	Chloroplast
BnaC04G0479000ZS	*BnIPT21*	464	52.46	5.79	-0.548	Chloroplast
BnaC06G0312200ZS	*BnIPT22*	332	37.45	9.07	-0.329	Chloroplast/ Cytoplasm/ Mitochondria
BnaC07G0117300ZS	*BnIPT23*	332	37.17	8.90	-0.212	Chloroplast
BnaC08G0418800ZS	*BnIPT24*	332	37.56	8.76	-0.172	Chloroplast
BnaC09G0474600ZS	*BnIPT25*	334	37.94	6.56	-0.215	Chloroplast

Bnascaffold0026G0006100ZS	*BnIPT26*	464	52.29	7.96	-0.447	Chloroplast
