# Supplementary figures and images for: Identification and Expression Analysis of the Isopentenyl Transferase (IPT) Gene Family under Lack of Nitrogen Stress in Oilseed (Brassica napus L.)
Source: Plants (Basel). 2023 May 30;12(11):2166. doi: 10.3390/plants12112166 (PMC10255845; doi:10.3390/plants12112166)

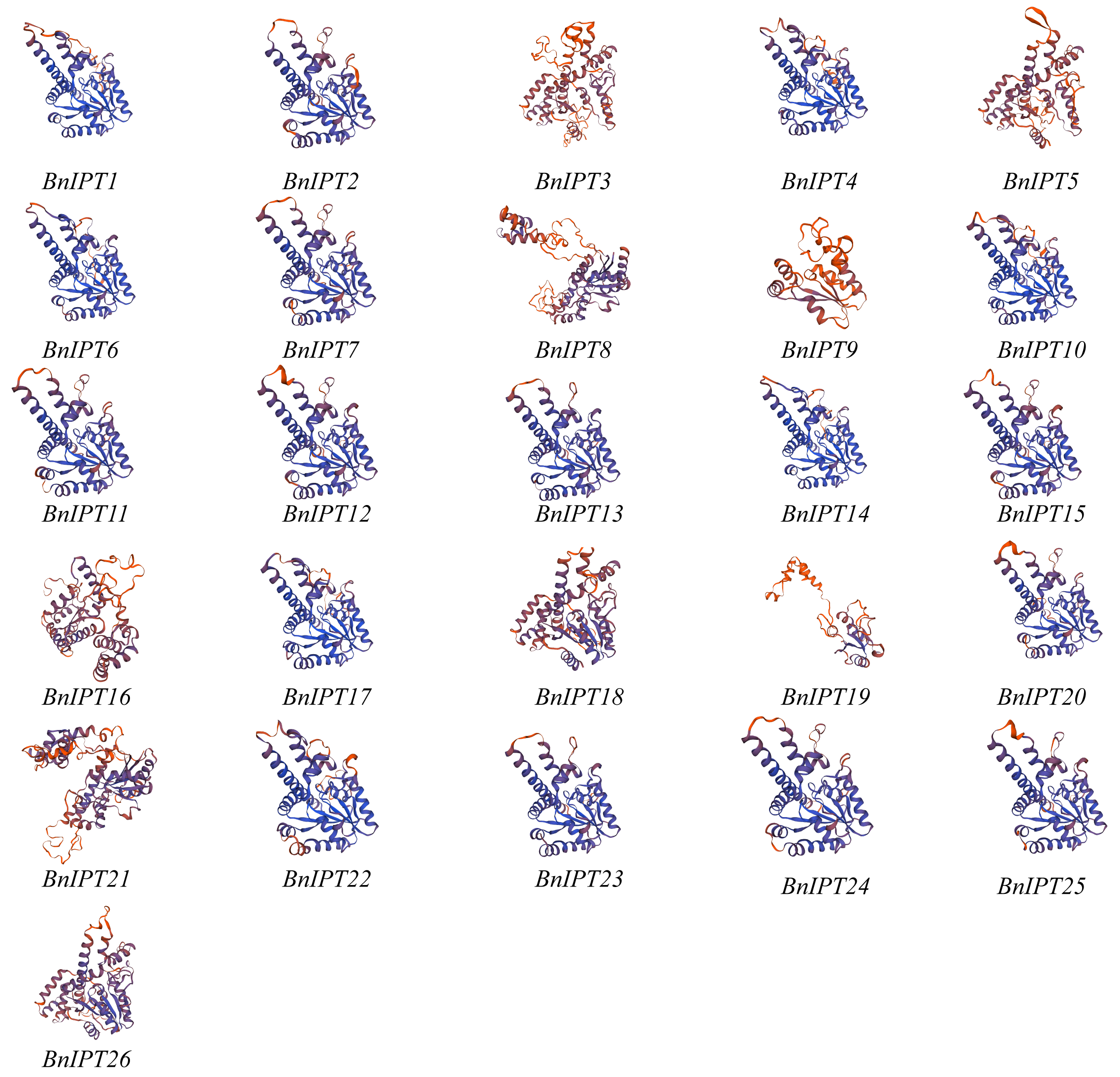

Supplement: Supplementary file 1 [file plants-12-02166-s001.zip › Figure S1.tif]
